# Pharmacological Insights into Optimal Dosing in Burning Mouth Syndrome: A Narrative Review of the Non-Linear Actions of Amitriptyline and Aripiprazole

**DOI:** 10.3390/jcm14207282

**Published:** 2025-10-15

**Authors:** Takahiko Nagamine

**Affiliations:** 1 Department of Psychiatric Internal Medicine, Sunlight Brain Research Center, Hofu 747-0066, Japan; anagamine@yahoo.co.jp; Tel.: +81-3-5803-5909; 2 Department of Psychosomatic Dentistry, Graduate School of Medical and Dental Sciences, Institute of Science, Bunkyo, Tokyo 113-8510, Japan

**Keywords:** burning mouth syndrome, amitriptyline, aripiprazole, dose-response, personalized medicine

## Abstract

**Background**: Burning Mouth Syndrome (BMS) is a nociplastic pain condition characterized by altered central nervous system pain processing, significantly impacting patient quality of life. Pharmacological management often involves amitriptyline (monotherapy) and aripiprazole (for refractory cases) in Japan. However, the therapeutic efficacy of these drugs in BMS frequently exhibits a non-sigmoid (U-shaped or bell-shaped) dose–response relationship, indicating a clinically effective dose that is often considerably lower than those used for their primary indications and challenging conventional pharmacological assumptions. **Method**: This paper synthesizes existing pharmacological knowledge to elucidate the mechanisms underlying the non-dose-dependent actions of amitriptyline and aripiprazole in BMS. It focuses on their specific interactions with key neurotransmitter systems and receptors, particularly N-methyl-D-aspartate (NMDA) receptors and dopamine D2 receptors, to explain the observed non-linear dose–response and the importance of identifying a personalized therapeutic window. **Result**: Amitriptyline demonstrates efficacy in BMS at low doses (e.g., 25 mg), primarily through its action as an NMDA receptor antagonist via calcium-dependent desensitization and open-channel block, addressing central sensitization. Its effects are distinct from its antidepressant actions, and the “serotonin paradox” highlights the complexity of serotonin’s role in pain. Aripiprazole, utilized for refractory BMS, acts as a dopamine D2 receptor partial agonist, leading to a non-linear dose–response where sustained therapeutic effect is observed at specific low doses (e.g., 1.7–1.8 mg/day). This non-linearity is attributed to partial agonism, alongside interactions with serotonin 5-HT1A and 5-HT2A receptors. The general non-dose-dependency for both drugs is further explained by phenomena such as multiple binding sites with differing affinities, receptor desensitization/downregulation, activation of counter-regulatory mechanisms, and hormesis. **Discussion**: The observed non-linear dose–response curves for amitriptyline and aripiprazole in BMS underscore the inadequacy of a “one-size-fits-all” treatment approach. This necessitates a shift towards personalized medicine, which considers individual patient factors including pharmacogenomics, comorbidities, age, organ function, and psychological/social profiles. The true “personalized therapeutic window” is a balance between achieving significant pain relief and minimizing adverse effects, emphasizing careful titration and patient-centered care. **Conclusions**: The pharmacological actions of amitriptyline and aripiprazole in BMS are not linearly dose-dependent, but rather exhibit a personalized therapeutic window driven by complex interactions with NMDA and D2 receptors and adaptive physiological responses. This intricate pharmacological landscape mandates a personalized medicine approach to optimize treatment outcomes, improve patient adherence, and enhance the quality of life for individuals suffering from this challenging nociplastic pain condition.

## 1. Introduction

Burning Mouth Syndrome (BMS) is a chronic intraoral pain condition characterized by a burning sensation, often bilateral, in the absence of identifiable mucosal lesions or systemic disease [1]. It significantly impacts patients’ quality of life, leading to discomfort, sleep disturbances, anxiety, and depression. Unlike nociceptive pain, which arises from actual or threatened tissue damage, or neuropathic pain, which results from nerve injury, BMS is classified as a nociplastic pain condition [2]. This designation indicates that the pain originates from altered nociception in the central nervous system (CNS), involving complex changes in the processing of pain signals, most notably central sensitization. Central sensitization is characterized by an amplification of pain signals within the CNS, leading to heightened sensitivity to pain (hyperalgesia) and the experience of pain from non-painful stimuli (allodynia). The N-methyl-D-aspartate (NMDA) receptor, a crucial ionotropic glutamate receptor, is thought to play a central role in the molecular mechanisms underlying central sensitization and the chronification of pain [3].

Pharmacological interventions for BMS often involve off-label use of medications primarily developed for psychiatric disorders, such as antidepressants and atypical antipsychotics. In Japan, amitriptyline monotherapy is the most commonly employed drug treatment for BMS [4], with aripiprazole being increasingly utilized in combination therapy for refractory cases [5]. However, a notable observation in the clinical management of BMS with these drugs is that their therapeutic efficacy does not always follow a conventional dose-dependent linear or sigmoid curve [6]. Instead, studies and clinical experience suggest a non-sigmoid (U-shaped or bell-shaped) dose–response relationship, indicating the existence of a clinically effective dose that is often considerably lower than the doses typically used for their primary indications [7]. This phenomenon challenges traditional pharmacological assumptions and underscores the critical need for personalized medicine in the treatment of BMS. This paper aims to explore the intricate mechanisms behind the non-dose-dependent pharmacological actions of amitriptyline and aripiprazole in BMS. We will delve into their specific interactions with neurotransmitter systems and receptors, particularly NMDA and dopamine D2 receptors, which are important receptors as therapeutic targets in BMS [8], to explain why a personalized therapeutic window, rather than maximal dose, is paramount for achieving sustained therapeutic benefits and improving treatment continuation rates. Furthermore, we will emphasize how these insights pave the way for a more individualized approach to managing this complex and debilitating condition. The discussion will unify the underlying mechanisms for both drugs—NMDA antagonism and D2 partial agonism—under the framework of hormesis to provide a clear rationale for the non-linear pharmacodynamics observed in BMS treatment.

## 2. Search Strategy and Synthesis Approach

The objective of this narrative review is to synthesize existing knowledge on the pharmacological mechanisms of amitriptyline and aripiprazole in BMS and explain their non-linear dose–response relationship. A comprehensive literature search was conducted to identify relevant studies and reviews in pharmacology, neurobiology, and clinical medicine. The search strategy was executed across major biomedical databases, including PubMed, Scopus, and the Cochrane Library. The search queries were constructed using a combination of keywords and Medical Subject Headings (MeSH) to ensure broad coverage of the topic. The primary search terms included “Burning Mouth Syndrome,” “BMS,” “amitriptyline,” “aripiprazole,” “dose-response,” “nociplastic pain,” “central sensitization,” “NMDA receptor,” and “dopamine D2 receptor.” These terms were combined using Boolean operators (“AND,” “OR”) to refine the search. Initial screening involved reviewing titles and abstracts to identify studies and reviews relevant to the pharmacological mechanisms and dose–response characteristics of these drugs in BMS or other chronic pain conditions. The literature search was not exhaustive or systematic in the manner of a PRISMA-compliant review but was designed to provide a broad, mechanistic narrative synthesis.

The inclusion criteria for full-text review were (1) papers published in English, (2) clinical studies, case reports, and review articles, (3) articles discussing the pharmacological actions of amitriptyline or aripiprazole, and (4) content explaining the dose–response relationship of these drugs, especially in the context of pain management or their non-psychiatric uses. Exclusion criteria included articles not available in full text, studies focused solely on the primary psychiatric indications of these drugs without relevance to pain, and papers that were not peer-reviewed. The identified literature was then meticulously analyzed to extract key findings related to the mechanisms of action, receptor interactions, and dose–response curves. The final synthesis was structured to build a coherent narrative that explains the complexity of BMS pharmacology and the rationale for personalized medicine. All statistical data, such as values and confidence intervals, discussed in this review are extracted directly from the cited primary literature.

## 3. The Nuanced Pharmacological Landscape of BMS Treatment: Beyond Simple Dose-Dependency

**Figure 1:**
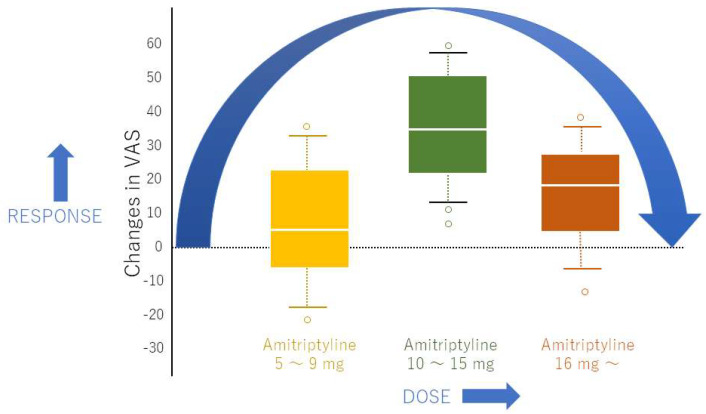
Dose–response relationship of amitriptyline for burning mouth syndrome. The diagram was created based on Nagamine, T. 2024 [10].

Traditional pharmacological models often assume a straightforward relationship between drug dose and therapeutic effect, where increasing drug concentration leads to a proportionally increasing effect until a plateau or saturation is reached, typically depicted as a sigmoid dose–response curve [9]. However, for medications like amitriptyline and aripiprazole in the treatment of BMS, the observed relationship between dose and therapeutic efficacy, particularly in terms of treatment continuation, often deviates significantly from this simple model [7]. Instead, clinical data suggest non-sigmoid, often U-shaped or bell-shaped, curves (Figure 1) [10]. This indicates the presence of a personalized therapeutic window that does not necessarily correspond to the highest tolerated dose. Importantly, for amitriptyline, this clinically effective dose is considerably lower than the doses typically required for its antidepressant effects, highlighting a dissociation between its primary psychiatric indication and its analgesic properties in nociplastic pain.

This non-linear dose–response poses significant challenges and opportunities in the management of BMS [10]. It implies that simply escalating the dose in pursuit of greater efficacy may not only be ineffective but could also increase the likelihood of adverse effects, leading to premature treatment discontinuation. Understanding the underlying mechanisms that contribute to this non-dose-dependent phenomenon is crucial for optimizing therapeutic outcomes in BMS.

## 4. Amitriptyline: Complex NMDA Receptor Modulation and the Serotonin Paradox in BMS

Amitriptyline, a tricyclic antidepressant (TCA), has long been a cornerstone in the pharmacological management of chronic pain, including BMS, even in the absence of a diagnosed mood disorder. Its efficacy in mitigating BMS symptoms is often observed at remarkably low doses (e.g., 25 mg/day or even lower), which typically do not elicit its characteristic antidepressant effects [4]. This observation suggests that its therapeutic benefits in BMS extend beyond, and are likely distinct from, its conventional actions as an antidepressant.

While antidepressants, including TCAs, are known to influence neurotransmitters such as serotonin and norepinephrine, which are involved in the regulation of pain signaling through the potentiation of descending inhibitory pain pathways, their role in BMS is more intricate. These pathways, originating in brainstem regions like the periaqueductal gray and rostral ventromedial medulla, utilize serotonin and norepinephrine to inhibit pain signal transmission at the spinal cord level. By inhibiting the reuptake of both serotonin and norepinephrine, amitriptyline increases the availability of these neurotransmitters in the synaptic cleft, theoretically enhancing endogenous pain inhibition [11]. However, the role of serotonin in pain is far from straightforward. While serotonin has been observed to exhibit analgesic properties, it can paradoxically contribute to the experience of pain in certain instances, a phenomenon often referred to as the “serotonin paradox” [12]. The human body possesses seven distinct types of serotonin receptors (5-HT1 to 5-HT7) with multiple subtypes, and their functional distinctions in modulating nociceptive transmission remain a subject of ongoing research. For instance, 5-HT7 spinal receptors have been shown to modulate pain through gamma-aminobutyric acid (GABA) interneurons, potentially exerting an inhibitory effect. Conversely, neurotransmission via 5-HT2 and 5-HT3 receptors has been shown to promote pain [13]. This complexity implies that the overall effect of serotonin modulation by amitriptyline can be highly dependent on the specific serotonin receptor subtypes activated, and at higher doses, unintended activation of pro-nociceptive serotonin receptors might occur, contributing to adverse effects or diminished pain relief. This phenomenon is increasingly recognized in the treatment of various chronic pain conditions, not just BMS, suggesting that low-dose TCAs achieve a delicate balance of neuromodulation. This complex interplay suggests that the efficacy of antidepressants in BMS is likely attributable to factors beyond a simple antidepressant effect or a uniform activation of descending pain inhibitory pathways.

Beyond its classical neurotransmitter reuptake inhibition, amitriptyline possesses additional pharmacological properties that are highly relevant to its analgesic effects in nociplastic pain. It can block sodium channels, similar to local anesthetics, thereby stabilizing neuronal membranes and reducing aberrant neuronal firing characteristic of neuropathic and nociplastic pain [14]. More critically for BMS, which is thought to involve central sensitization mediated by NMDA receptor changes, amitriptyline functions as an NMDA receptor antagonist through two distinct molecular mechanisms [15]. Firstly, amitriptyline has been shown to enhance the calcium-dependent desensitization of NMDA receptors. In the presence of amitriptyline, these receptors exhibit diminished responsiveness to glutamate, especially in circumstances where intracellular calcium levels are elevated. This is particularly significant in central sensitization, where sustained neuronal activity can lead to increased intracellular calcium, contributing to NMDA receptor hyperexcitability. By promoting desensitization, amitriptyline effectively dampens this heightened responsiveness. Secondly, amitriptyline has been demonstrated to function as a trapping open-channel blocker of NMDA receptors [15]. This effect is attributed to the ability of amitriptyline to bind to the receptor’s ion channel only when it is in the open state, thereby physically impeding the flow of ions (such as calcium and sodium) through the channel. Once bound, the drug can become “trapped” within the channel, providing sustained blockade even after the drug concentration in the extracellular space declines. This multifaceted interaction with NMDA receptors is a crucial mechanism by which amitriptyline reduces the excitability of neurons involved in pain pathways, directly addressing the underlying pathophysiology of central sensitization in BMS [8,16]. This intricate interaction with NMDA receptors likely contributes significantly to its non-linear dose–response, where an effective low dose achieves sufficient NMDA receptor modulation without inducing excessive blockade or off-target effects.

## 5. Aripiprazole: Dopaminergic Modulation and Partial Agonism in Refractory BMS

While amitriptyline remains the first-line pharmacological agent for BMS in Japan, atypical antipsychotics like aripiprazole are gaining attention, particularly as an augmentation strategy for refractory cases or those with significant psychological overlay [17]. Their mechanisms in chronic pain management are distinct from antidepressants and are notably complex.

**Figure 2:**
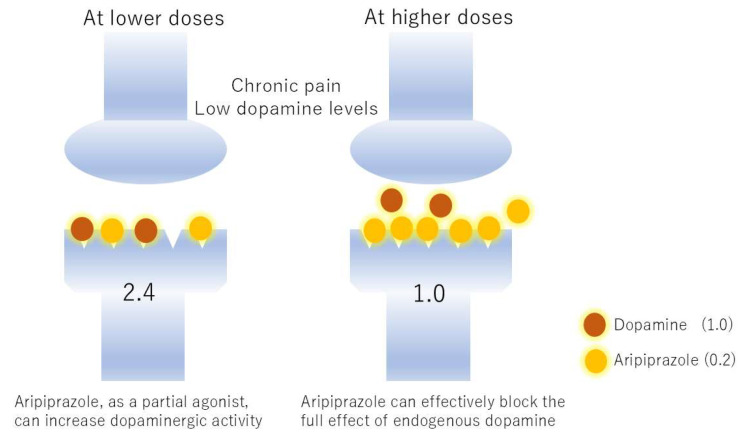
Reversal of the effects of aripiprazole by different dopamine concentrations. Suppose dopamine transmits a stimulus of 1.0 and aripiprazole transmits 0.2. In chronic pain, dopamine function is reduced, setting the dopamine concentration to 2. At low doses, aripiprazole supplements dopamine transmission, resulting in a transmission of 2.4. However, at high doses, aripiprazole pushes aside the small amount of dopamine, occupying the receptors and reducing transmission to 1.0. The former is an agonist effect and the latter is an antagonist effect.

Aripiprazole’s primary pharmacological action is its partial agonism at dopamine D2 receptors. The dopamine system plays a critical and multifaceted role in pain perception, reward pathways, and motivation [18]. Dysregulation of dopamine has been implicated in various chronic pain states, particularly those with features of anhedonia, fatigue, or cognitive dysfunction [19]. By stabilizing dopaminergic signaling, aripiprazole may influence the affective and motivational components of pain, potentially reducing pain-related distress and improving coping mechanisms [20]. The concept of partial agonism is crucial to understanding aripiprazole’s non-linear dose–response. A partial agonist binds to a receptor and produces a submaximal response, even when all receptors are occupied. Importantly, the effect of a partial agonist is dependent on the baseline level of endogenous ligand (in this case, dopamine) [21]. At lower doses, where endogenous dopamine levels might be insufficient or dysregulated in certain pain states, aripiprazole, as a partial agonist, can increase dopaminergic activity by providing some level of agonism. However, at higher doses, if endogenous dopamine levels are high, aripiprazole can effectively block the full effect of endogenous dopamine by competitively binding to the D2 receptors, thereby acting more like an antagonist and reducing overall dopaminergic activity (Figure 2) [22]. This dual nature of partial agonism—acting as a weak agonist in low-dopamine states and an antagonist in high-dopamine states—inherently leads to a complex, non-linear dose–response relationship. For aripiprazole in BMS, sustained treatment continuation rates have been observed at specific low doses (e.g., 1.7–1.8 mg/day) [7]. Doses higher than this effective range may lead to excessive D2 receptor blockade or other compensatory mechanisms, resulting in diminished returns or increased discontinuation due to side effects.

Beyond its D2 receptor activity, aripiprazole also has partial agonism at serotonin 5-HT1A receptors and antagonism at 5-HT2A receptors [23]. These actions further contribute to its complex neurochemical profile and can influence mood, anxiety, and potentially pain processing pathways. The 5-HT1A receptor is involved in anxiety and depression, and its partial agonism could contribute to anxiolytic effects that indirectly benefit BMS patients by reducing stress-exacerbated symptoms. The 5-HT2A antagonism might modulate pain processing, especially given the “serotonin paradox” where some serotonin receptors promote pain [12]. Similar to antidepressants, emerging research suggests that some atypical antipsychotics may possess anti-inflammatory properties, modulating cytokine production and reducing neuroinflammation, which is increasingly recognized as a contributor to chronic pain. They may also exert neuroprotective effects, potentially influencing neuronal plasticity and reducing maladaptive changes in the central nervous system associated with pain chronification [24]. While more research is needed specifically in the context of BMS, these additional mechanisms could contribute to the overall therapeutic profile and the complex dose–response of aripiprazole.

## 6. Mechanisms Explaining Non-Dose-Dependency

The observed non-sigmoid (U-shaped or bell-shaped) dose–response relationships for amitriptyline and aripiprazole in BMS treatment continuation are critical findings that challenge conventional pharmacological assumptions and strongly support the need for individualized dosing. Several interconnected pharmacological phenomena can account for such complex relationships.

### 6.1. Multiple Binding Sites/Targets with Different Affinities

Many psychotropic medications, particularly those with broad pharmacological profiles like tricyclic antidepressants and atypical antipsychotics, interact with various molecular targets beyond their primary mechanisms, and each target can have different binding affinities [25]. A drug might exert its therapeutic effect by binding to high-affinity targets at lower concentrations. As the dose increases, these high-affinity sites become saturated, and the drug begins to bind to lower-affinity targets. These lower-affinity targets might be associated with adverse effects, off-target actions, or even counter-therapeutic effects that diminish the overall positive response, leading to a decline in efficacy or increased side effects and patient discontinuation [25]. For instance, amitriptyline, in addition to its effects on serotonin and norepinephrine reuptake and direct sodium channel blockade, robustly inhibits NMDA receptors through two distinct pharmacological actions (calcium-dependent desensitization and open-channel block) [15]. At lower concentrations, it might preferentially bind to high-affinity sites within the NMDA receptor complex or other key pain-modulating targets that mediate the desired analgesic effect. However, as the dose increases, it might begin to bind to lower-affinity sites that lead to undesirable anticholinergic effects, antihistaminic effects (e.g., sedation), or even excessive NMDA blockade that could paradoxically impair beneficial synaptic plasticity.

Similarly, aripiprazole, despite its primary action as a D2 partial agonist, also interacts with various serotonin receptors (e.g., 5-HT1A partial agonism, 5-HT2A antagonism) and other systems [22]. The personalized therapeutic balance of these multiple interactions for pain relief and treatment continuation might occur within a narrow concentration range. Beyond this, saturation of beneficial targets and engagement of lower-affinity, adverse-effect-producing targets could lead to a U-shaped response.

### 6.2. Receptor Desensitization or Downregulation

Another key mechanism contributing to non-linear dose–response curves is the adaptive response of target receptors to excessive or prolonged stimulation [26]. When receptors are continuously exposed to high concentrations of an agonist, the cell can initiate feedback mechanisms to protect itself from overstimulation. These mechanisms can include desensitization and downregulation. Desensitization is a rapid decrease in the responsiveness of the receptor, even in the continued presence of the agonist. This can occur through phosphorylation of the receptor, leading to uncoupling from its signaling pathways, or internalization of the receptor from the cell surface. Downregulation is a slower, more prolonged reduction in the total number of receptors expressed on the cell surface. This involves decreased synthesis or increased degradation of receptor proteins.

For drugs acting on receptors, beyond a certain clinically effective concentration, increasing the drug dose might not lead to a proportionally greater effect, or could even lead to a reduced effect as the target system adapts negatively. This mechanism is highly relevant for aripiprazole, given its partial agonism at D2 receptors. While optimal partial agonism might be achieved at low doses (1.7–1.8 mg/day) to stabilize dopaminergic signaling, higher doses could lead to excessive D2 receptor occupancy, triggering desensitization or downregulation of these receptors [7]. This would paradoxically reduce the overall effectiveness of dopaminergic modulation, thereby diminishing treatment continuation due to perceived inefficacy or increased side effects. The intricate modulation of NMDA receptors by amitriptyline could also be subject to similar desensitization or compensatory mechanisms if the blockade is too profound or prolonged.

### 6.3. Activation of Counter-Regulatory Mechanisms

The human body possesses intricate homeostatic mechanisms designed to maintain physiological balance [27]. When a drug exerts an initial beneficial effect, it can sometimes trigger a compensatory or counter-regulatory response that attempts to restore the original pathological state. This “rebound” effect can manifest as the drug dose increases. At lower drug doses, the beneficial effects might outweigh the compensatory mechanisms. However, at higher drug doses, this compensatory mechanism might become overly active or overcompensate, leading to a diminished or even opposite effect compared to the desired therapeutic outcome. This can contribute significantly to treatment discontinuation as patients no longer perceive benefit or experience worsening symptoms.

For example, a beneficial dampening of central sensitization by amitriptyline at an effective low dose might, at higher doses, trigger a cascade of neuroplastic changes or neurotransmitter imbalances that counter the initial positive effect [15]. Similarly, optimal dopaminergic modulation by aripiprazole could, at higher doses, activate downstream compensatory pathways that lead to adverse effects or reduced pain relief [22]. The complexity of pain signaling networks, involving multiple excitatory and inhibitory pathways, makes them particularly susceptible to such counter-regulatory phenomena.

### 6.4. Hormesis

**Figure 3:**
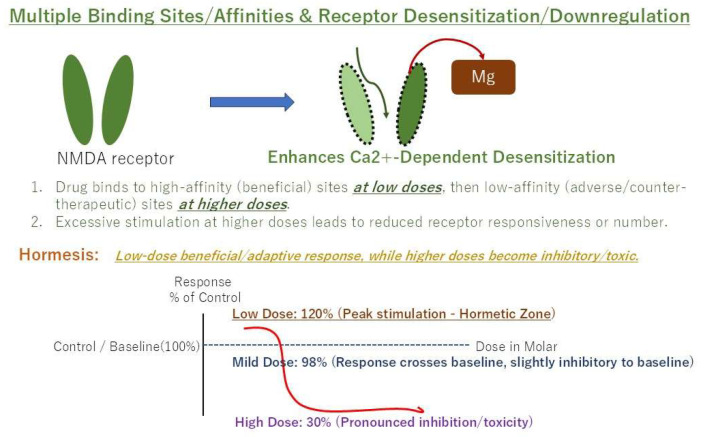
Example of a hormetic dose–response curve (U-shaped). Low doses are stimulatory/beneficial, while high doses are inhibitory/toxic.

Hormesis is a dose–response phenomenon characterized by a low-dose stimulation and a high-dose inhibition, resulting in a U-shaped or inverted U-shaped (bell-shaped) dose–response curve [28]. This concept provides a unifying framework for the non-linear pharmacodynamics of both amitriptyline and aripiprazole in BMS, as both mechanisms (NMDA antagonism and D2 partial agonism) operate in a manner consistent with low-dose adaptive signaling. In the context of pharmacology, it suggests that a substance that is toxic or inhibitory at high doses can elicit a beneficial, adaptive, or stimulatory response at low doses (Figure 3). For amitriptyline in BMS, a low dose (e.g., 5–10 mg/day initially, titrated to 25 mg/day effective dose) might initiate subtle pain modulation or adaptive changes within the CNS, perhaps through its partial agonism at specific NMDA receptor subunits or a very selective impact on certain pain pathways, or by stimulating neurotrophic factors that promote neuronal health and plasticity [10,14]. These low-dose effects might be adaptive and promote healing or rebalancing of the central pain processing system. However, if the dose is too low to achieve sustained clinical improvement, patients might discontinue due to perceived inefficacy. An intermediate dose (e.g., 25 mg/day for amitriptyline or 1.7–1.8 mg/day for aripiprazole) might hit a “personalized therapeutic window” where these adaptive responses and direct analgesic effects, including the complex effects on NMDA receptors and D2 receptors, are optimized without overwhelming the system or causing intolerable side effects. Beyond this window, higher concentrations engage inhibitory or adverse effects, leading to treatment decline.

## 7. Translational Implications and Limitations

Given the inherent heterogeneity of chronic pain, the significant variability in individual drug responses, and the compelling evidence of non-linear dose–response curves for key pharmacological agents in BMS, a “one-size-fits-all” approach to treatment is fundamentally insufficient [8]. It frequently leads to suboptimal outcomes, prolonged patient suffering, and increased healthcare costs due to trial-and-error prescribing, medication changes, and management of side effects. This complex pharmacological landscape makes personalized medicine not merely an ideal but an absolute necessity for effective BMS management. Personalized medicine in this context means tailoring treatment strategies to the individual patient, meticulously considering their unique biological, psychological, and social profile [29]. This holistic approach moves beyond symptom-focused prescribing to address the underlying mechanisms of pain in each patient and optimize their response to specific therapies. Individualized dosing strategies for BMS must therefore consider a multitude of factors beyond simple symptom assessment.

### 7.1. Pharmacogenomics

Genetic polymorphisms can significantly alter drug metabolism and receptor sensitivity. For instance, variations in cytochrome P450 (CYP) enzymes (e.g., CYP2D6, CYP2C19), which are crucial for metabolizing amitriptyline, can drastically alter drug clearance, leading to higher or lower effective plasma concentrations for a given dose [30]. Patients classified as “poor metabolizers” might experience exaggerated effects and increased side effects even at low doses, while “ultrarapid metabolizers” might require higher doses to achieve therapeutic levels, though this must be balanced with the non-linear response. Similarly, genetic variations in drug transporters or target receptors (like NMDA or D2 receptors) could influence drug efficacy and tolerability [31]. While currently not routine for BMS, pharmacogenomic testing holds immense promise for guiding initial drug selection and dosing to minimize trial-and-error.

### 7.2. Comorbidities and Polypharmacy

Patients with chronic pain, including BMS, often present with multiple comorbidities such as cardiovascular disease, diabetes, gastrointestinal disorders, and other psychiatric conditions (e.g., generalized anxiety disorder, depression). Consequently, they are frequently on multiple medications, leading to a complex web of potential drug–drug interactions [5]. These interactions can influence drug absorption, distribution, metabolism, and excretion, altering the effective plasma concentration of BMS medications. For example, co-administration of other drugs that inhibit or induce CYP enzymes involved in amitriptyline or aripiprazole metabolism can lead to unexpected increases or decreases in drug levels, necessitating careful dose adjustment [32]. A thorough medication reconciliation and an understanding of potential pharmacokinetic and pharmacodynamic interactions are vital.

### 7.3. Patient-Specific Physiological Factors

Individual physiological differences significantly influence drug pharmacokinetics and pharmacodynamics. Older adults, for instance, often have reduced metabolic capacity (e.g., decreased hepatic enzyme activity) and decreased renal clearance, coupled with an increased sensitivity to CNS-acting medications. This necessitates lower starting doses and slower titration rates, as highlighted by the efficacy of low-dose amitriptyline in elderly BMS patients. Impaired organ function can significantly affect drug clearance, leading to drug accumulation and increased risk of adverse effects. Dosing adjustments are often necessary in these patient populations.

The specific characteristics of the pain itself can influence therapeutic response. Some studies suggest that greater initial pain intensity or an irregular pain pattern might predict a better response to certain agents, although further research is needed to fully elucidate these relationships in the context of personalized dosing for amitriptyline and aripiprazole [4,5]. The patient’s psychological state (e.g., anxiety levels, presence of depression, catastrophizing thoughts, pain beliefs), social support networks, and cultural background can profoundly influence their perception of pain, adherence to treatment, and reported outcomes. While not directly influencing drug pharmacokinetics, these factors dictate how a patient experiences side effects, perceives treatment efficacy, and ultimately, whether they continue with a given therapy. A seemingly optimal pharmacological dose might be rendered ineffective if the patient’s psychological distress or poor coping mechanisms lead to non-adherence or a heightened perception of residual pain. Integrating psychological support and pain education into the treatment plan is therefore essential for optimizing the overall therapeutic outcome.

The “optimal” dose is ultimately subjective and dynamic, requiring a delicate balance between achieving significant pain relief and maintaining an acceptable side effect profile for that individual patient. A dose that provides substantial symptom improvement but causes intolerable side effects (e.g., severe dry mouth, sedation, cognitive impairment with amitriptyline; akathisia, restlessness with aripiprazole) is not truly optimal for that patient, as it will inevitably lead to non-adherence and treatment discontinuation. This underscores the need for careful titration, patient education, and shared decision-making, allowing patients to actively participate in finding their individual therapeutic window. For BMS, where altered central pain processing specifically implicates NMDA and D2 receptors as key targets, understanding the nuanced actions of drugs like amitriptyline and aripiprazole on these systems is crucial. The observation that treatment continuation rates can be highest at specific, often low, doses, and decline with further dose escalation, provides compelling evidence that simple dose titration is often counterproductive. While currently limited for routine clinical use in BMS, future research incorporating biomarkers (e.g., inflammatory markers, genetic markers related to pain pathways) and functional neuroimaging (e.g., SPECT, fMRI) could provide more objective insights into underlying pain mechanisms and predict treatment response, further refining personalized approaches. This could specifically focus on the activity and plasticity of NMDA and D2 receptors in the context of BMS to guide treatment selection and optimization. To summarize, Table 1 shows the effects of amitriptyline and aripiprazole on BMS.

## 8. Conclusions

The pharmacological actions of amitriptyline and aripiprazole in BMS are not linearly dose-dependent, but rather exhibit a personalized therapeutic window driven by complex interactions with NMDA and D2 receptors and adaptive physiological responses, which are consistent with the principles of hormesis. This intricate pharmacological landscape, characterized by multiple binding sites and a switch from beneficial low-dose modulation to detrimental high-dose inhibition/antagonism, mandates a personalized medicine approach. This approach must prioritize slow titration, careful side effect monitoring, and acknowledgment of patient-specific factors (e.g., pharmacogenomics) to optimize treatment outcomes, improve patient adherence, and enhance the quality of life for individuals suffering from this challenging nociplastic pain condition.

## Figures and Tables

**Table 1:** Comparison of amitriptyline and aripiprazole pharmacodynamics in burning mouth syndrome (BMS).

	Amitriptyline in BMS	Aripiprazole in BMS
Primary Indication in BMS	First-line monotherapy in Japan	Augmentation for refractory cases
Primary Proposed Analgesic Mechanism	NMDA Receptor Antagonist (Open-channel block and enhanced calcium-dependent desensitization)	Dopamine D2 Receptor Partial Agonist (Stabilization of dopaminergic signaling)
Other Key Receptor Interactions	Serotonin (5-HT) and Norepinephrine Reuptake Inhibitor; Sodium Channel Blockade; Anticholinergic (M1) and Antihistaminic (H1) effects	Serotonin 5-HT1A Partial Agonist; 5-HT2A Antagonist
Clinically Effective Dose Range	Low dose, e.g., 10–25 mg/day	Very low dose, e.g., 1.7–5 mg/day (often 1.8 mg/day for optimal continuation)
Dose-Limiting Adverse Effects (Higher Dose)	Anticholinergic effects (dry mouth, constipation), sedation, cardiotoxicity	Akathisia, sedation, increased anxiety (potentially due to excessive D2 antagonism)
Dose–Response Profile	Non-linear: often U-shaped/bell-shaped (efficacy drops at higher doses)	Non-linear: bell-shaped (due to partial agonism switching to functional antagonism)

## Data Availability

The data that support the findings of this study are available from the corresponding author upon reasonable request.

## Abbreviations

The following abbreviations are used in this manuscript:

BMS	Burning Mouth Syndrome
CNS	central nervous system
CYP	cytochrome P450
GABA	gamma-aminobutyric acid
NMDA	N-methyl-D-aspartate
TCA	tricyclic antidepressant
